# Dynamic changes of Chest CT follow-up in Coronavirus Disease-19 (COVID-19) pneumonia: relationship to clinical typing

**DOI:** 10.1186/s12880-020-00491-2

**Published:** 2020-08-05

**Authors:** Nian Liu, Guanghong He, Xiongxiong Yang, Jianxin Chen, Jie Wu, Min Ma, Wenying Lu, Qiang Li, Tao Cheng, Xiaohua Huang

**Affiliations:** 1 Department of Radiology, Affiliated Hospital of North Sichuan Medical College, No.63 Wenhua Road, Shunqing District, Nanchong, 637000 China; 2 Department of Radiology, Nanchong Hospital of Traditional Chinese Medicine, Nanchong, 637000 China; 3 Department of Radiology, West China-Guang’an Hospital, Sichuan University, Guangan, 638000 China; 4 Department of CT/MRI, Nanchong Central Hospital, Nanchong, 637000 China; 5 Department of Radiology, Wusheng People’s Hospital, Guangan, 638000 China; 6 Department of Radiology, Langzhong People’s Hospital, Nanchong, 637000 China; 7 Department of Radiology, Yuechi People’s Hospital, Guangan, 638000 China

**Keywords:** COVID-19, Coronavirus infections, Pneumonia, CT, Follow-up studies

## Abstract

**Background:**

To investigate the CT changes of different clinical types of COVID-19 pneumonia.

**Methods:**

This retrospective study included 50 patients with COVID-19 from 16 January 2020 to 25 February 2020. We analyzed the clinical characteristics, CT characteristics and the pneumonia involvement of the patients between the moderate group and the severe and critical group, and the dynamic changes of severity with the CT follow-up time.

**Results:**

There were differences in the CT severity score of the right lung in the initial CT, and total CT severity score in the initial and follow-up CT between the moderate group and the severe and critical group (all *p* < 0.05). There was a quadratic relationship between total CT severity score and CT follow-up time in the severe and critical group (r^2^ = 0.137, *p* = 0.008), the total CT severity score peaked at the second follow-up CT. There was no correlation between total CT severity score and CT follow-up time in the moderate group (*p* > 0.05). There were no differences in the occurrence rate of CT characteristics in the initial CT between the two groups (all *p* > 0.05). There were differences in the occurrence rate of ground-glass opacity and crazy-paving pattern in the second follow-up CT, and pleural thickening or adhesion in the third follow-up CT between the two groups (all *p* < 0.05).

**Conclusions:**

The CT changes of COVID-19 pneumonia with different severity were different, and the extent of pneumonia involvement by CT can help to assess the severity of COVID-19 pneumonia rather than the initial CT characteristics.

## Background

Since the outbreak of coronavirus disease 2019 (COVID-19) pneumonia in Wuhan, Hubei Province of China in December 2019, with the increase in the number of global cases, COVID-19 has posed major threats to the public health [1, 2]. With the joint efforts of the World Health Organization (WHO), clinicians and scientists, professional consensus, guidelines, and management systems have gradually established to prevent transmission and promote diagnosis and treatment [3–5].

Chest CT is one of the significant methods for screening and diagnosis of COVID-19 pneumonia [6–8]. Chest CT may be helpful for early diagnosis, especially when a person is suspected with COVID-19, but reverse transcription-polymerase chain reaction (RT-PCR) screening is negative [9–12]. The initial CT manifestation of COVID-19 pneumonia had certain regularity and specificity [3, 4, 13–15], which provided an important basis for diagnosis and treatment. In the present, the National Health Commission of the People’s Republic of China [16] has taken the CT manifestations of pneumonia absorption as one of the discharge criteria. Chest CT follow-up is of great value in the evaluation of the condition assessment and therapeutic effect of COVID-19 pneumonia [17–19]. However, it is not clear whether there are differences in chest CT findings of COVID-19 pneumonia with different severity. CT imaging is helpful to judge the evolution and prognosis of COVID-19 pneumonia, but whether COVID-19 with different severity has characteristic CT findings is still unclear. Therefore, it is still a question worth discussing whether CT imaging can help to evaluate the clinical type of COVID-19.

To answer that question, we retrospectively characterize the initial CT and follow-up CT findings in COVID-19 pneumonia with different clinical types, and the dynamic changes of different clinical types with the CT follow-up time from initial diagnosis until patient recovery. To our knowledge, this is a relatively comprehensive study of the characteristics and dynamic changes of CT between the moderate type and severe or critical type patients with COVID-19 pneumonia. This will help to clarify the value of CT in assessing the severity of COVID-19, and it also help clinicians and radiologists to improve the understanding of CT changes with the evolution of COVID-19 pneumonia of different severity.

## Methods

Our institutional review board approved this retrospective study and written informed consent from all patients was waived.

### Patients and CT image data acquisition

Fifty-three patients with confirmed COVID-19 from seven hospitals in Sichuan province in China were enrolled in our study. They underwent chest CT examination and reexamination from 16 January 2020 to 25 February 2020. Patient selection was consecutive, and the exclusion criteria were COVID-19 patients without abnormal manifestations on CT. Finally, three patients were excluded because of no abnormal manifestations on CT, and fifty patients were included. The clinical history, laboratory, epidemic characteristics, and chest CT images were collected. The date of both the initial chest CT examination and the first positive RT-PCR test was recorded for each participant. All patients underwent CT scanning and laboratory tests when the initial mouth swab test was performed. All patients were followed up with chest CT during the study period without intravenous contrast agents. All patients were imaged with 0.6 mm to 1.25 mm thick slices in commercial multi-detector CT scanners (SOMATOM Definition AS and STRATON MX, Siemens Healthineers, Erlangen, Germany; BrightSpeed scanner, GE Medical Systems, Milwaukee, Wis; Philips Ingenuity Core128, Philips Medical Systems, Best, Netherlands; and UCT 760 scanner, United Imaging, Shanghai, China). The mean CTDIvol was 6.6 ± 2.3 mGy (range: 4.1–10.3 mGy).

### Clinical evaluation

All patients were at least two positive results by real-time RT-PCR assay for COVID-19 at laboratory testing of respiratory secretions obtained from the nasopharyngeal swab, oropharyngeal swab, endotracheal aspirate, or bronchoalveolar lavage [3]. According to the guideline of COVID-19 (Trial Version 6) [16], the patients were typed into two groups. The moderate group was defined as: the patient had a fever, respiratory symptoms, and abnormal imaging findings of pneumonia. The severe and critical group was defined as meeting any of the following [20]: Severe respiratory distress (respiratory rate > 30 breaths/min); Oxygen saturation (SpO2) ≤ 93% at rest; Partial arterial oxygen pressure (PaO2)/ Fraction of inspired oxygen (FiO2) ≤ 300 mmHg (1 mmHg = 0.133 kPa); Respiratory failure and requirement for mechanical ventilation; Shock; and Combined with multi-organ failure and requirement for intensive care unit (ICU).

The patients with diagnosed COVID-19 pneumonia were isolated and hospitalized for treatment. Discharge standards [3, 16] are as follows: 1. The temperature returned to normal for more than 3 days; 2. Respiratory symptoms were significantly improved; 3. Pulmonary imaging showed obvious signs of absorption in acute exudation inflammation of the lungs; and 4. Respiratory nucleic acid was negative for two consecutive times (at least one-day sampling time interval). The patient who meets the four conditions simultaneously can be released from isolation.

### Chest CT evaluation

The initial and follow-up chest CT images were assessed as the following ten characteristics according to the Fleischner Society Glossary [21, 22] and peer-reviewed literature on viral pneumonia [13, 17], such as ground-glass opacity (GGO), crazy-paving pattern, consolidation, pleural thickening or adhesion, fibrosis, discrete nodules, cavitation, lymph node enlargement, pleural effusion, and bronchiectasis. A semi-quantitative scoring system was used to quantitatively estimate the extent of pulmonary involvement [13]. The area of abnormal pulmonary involvement was scored for each of the five lung lobes as follow: zero scores (no abnormal involvement), one score (1–25% abnormal involvement), two scores (26–49% abnormal involvement), three scores (50–75% abnormal involvement), and four scores (76–100% abnormal involvement). Finally, the total CT severity score was a sum of 5 lobe scores ranging from 0 to 20. The interval time between initial CT and the first follow-up CT scan was defined as Interval-1, and the interval between initial CT and the second follow-up CT was defined as Interval-2, and so on.

Image analysis was performed by two radiologists with more than 8 years of experience (N.L. and XH.H.) by using a DICOM Viewer software (Medixant. RadiAnt DICOM Viewer [Software]. URL: https://www.radiantviewer.com). Images were reviewed independently, and final scores were reached by consensus.

### Statistical analysis

All statistical analyses were conducted using SPSS software (version 22.0, U.S.A.). Quantitative variables were expressed as mean ± standard deviation (minimum-maximum) and the categorical variables were expressed as the percentage of the total. Demographic variables (e.g., age and gender), clinical characteristics (i.e., the hospitalized period and mean number of scans), and the occurrence rate of CT characteristics were compared by independent sample t test, chi-square test, or Fisher’s exact test. The Shapiro-Wilk test was used for the normal distribution. The CT severity scores were compared by the Mann-Whitney U test. SPSS curve estimation module was used to quantitatively evaluate the total CT severity score of pulmonary as a function of CT follow-up time. The statistical significance level was set at *p* = 0.05 with two-tailed.

## Results

### Demographic and clinical characteristics

The demographic and clinical characteristics of patients are listed in Table 1. There were 34 cases of the moderate group (mean age, 44 ± 12 years; age range, 21–69 years) and 16 cases of the severe and critical group (mean age, 50 ± 14 years; age range, 33–74 years). There was no significant difference in age, gender, and symptoms with fever and cough between the severe and critical group and the moderate group (all *p* > 0.05). There was a significant difference in exposure history between the severe and critical group and the moderate group (16/34 vs. 16/16, *p* < 0.001). The hospitalized period and numbers of scan in the severe and critical group were higher than that in the moderate group (28 ± 7 vs. 20 ± 8, *p* = 0.002; 6 ± 1 vs. 4 ± 1, *p* < 0.001, respectively). There was no significant difference in the consistency of PCR and CT results at the initial presentation between the two groups.

**Table 1:** Demographic and clinical characteristics

	All patients (*n* = 50)	Moderate type (*n* = 34)	Severe and critical type (*n* = 16)	t or χ^2^*P*-value
**Age (years)**	46 ± 13 (21–74)	44 ± 12 (21–69)	50 ± 14 (33–74)	0.080
**Gender**				0.952
Man	29	20	9	
Woman	22	15	7	
**Exposure history**				< 0.001^***^
Recent travel to Wuhan	32 (64%)	16 (47%)	16 (100%)	
Exposure to infected patient	18 (36%)	18 (53%)	0 (0%)	
**Symptoms**				
Fever	31 (62%)	20 (61%)	11 (69%)	0.500
Cough	29 (58%)	17 (50%)	12 (75%)	0.095
**Initial RT-PCR test and CT**				1.000
Both RT-PCR and CT positive	46 (92%)	31 (91%)	15 (94%)	
RT-PCR negative and CT positive	4 (8%)	3 (9%)	1 (6%)	
**The hospitalized period (d)**	23 ± 8 (10–43)	20 ± 8 (10–40)	28 ± 7 (19–43)	0.002^**^
**Mean number of scans**	4 ± 2 (2–8)	3 ± 1 (2–5)	6 ± 1 (3–8)	< 0.001^***^
**The interval between the adjacent scans (d)**	5 ± 3 (2–10)	5 ± 2 (2–13)	5 ± 2 (1–10)	NA

Note: Quantitative data were presented as mean ± standard deviation (minimum-maximum), while the counting data were presented as count (percentage of the total)

*NA* not applicable; ^**^p < 0.01; ^***^p < 0.001

### The findings of CT severity scores with CT follow-up time in the two groups

In the initial CT, the CT severity scores of right upper, middle and lower lobe in the severe and critical group was higher than that in the moderate group (1.7 ± 1.4 vs. 0.7 ± 0.5, 1.3 ± 1.3 vs. 0.4 ± 0.5, 1.9 ± 1.2 vs. 1.2 ± 0.7; all *p* < 0.05, Table 2). There were no significant differences in the CT scores of left upper and lower lobe between the two groups (all *p* > 0.05, Table 2). There were no significant differences in the number of involved lobes between the two groups (all *p* > 0.05). The total CT severity score of the severe and critical group in the initial CT and follow-up CT was significantly higher than that of the moderate group (all *p* < 0.05, Table 3). There were no significant differences in the interval time of Interval-1, Interval-2, Interval-3, and Interval-4 between the two groups (all *p* > 0.05, Table 3).

**Table 2:** Different findings of initial CT images between two clinical groups

	All patients (*n* = 50)	Moderate type (*n* = 34)	Severe and critical type (*n* = 16)	Mann-Whitney U or χ^2^*P*-value
**CT characteristics**				
ground glass opacity	44 (88%)	32 (94%)	12 (75%)	0.074
consolidation	24 (48%)	14 (41%)	10 (63%)	0.227
crazy-paving pattern	25 (50%)	17 (50%)	8 (50%)	1.000
pleural thickening or adhesion	26 (52%)	17 (50%)	9 (56%)	0.767
fibrosis	16 (32%)	10 (29%)	6 (38%)	0.746
discrete nodules	4 (8%)	3 (9%)	1 (6%)	1.000
bronchiectasis	3 (6%)	2 (6%)	1 (6%)	1.000
thoracic lymphadenopathy	2 (4%)	1 (3%)	1 (6%)	0.542
pleural effusion	2 (4%)	0 (0%)	2 (13%)	0.098
cavitation	1 (2%)	1 (3%)	0 (0%)	1.000
**Number of involved lobes**				
Right upper lobe	34 (68%)	21 (62%)	13 (81%)	0.208
Right middle lobe	24 (48%)	13 (38%)	11 (69%)	0.069
Right lower lobe	46 (92%)	31 (91%)	15 (94%)	1.000
Left upper lobe	34 (68%)	22 (74%)	12 (75%)	0.533
Left lower lobe	39 (78%)	24 (71%)	15 (94%)	0.080
**CT score in each lobe**				
Right upper lobe	1.0 ± 1.0 (0–4)	0.7 ± 0.5 (0–1)	1.7 ± 1.4 (0–4)	0.017^*^
Right middle lobe	0.7 ± 1.0 (0–4)	0.4 ± 0.5 (0–1)	1.3 ± 1.3 (0–4)	0.007^**^
Right lower lobe	1.5 ± 1.0 (0–4)	1.2 ± 0.7 (0–4)	1.9 ± 1.2 (0–4)	0.047^*^
Left upper lobe	1.0 ± 1.1 (0–4)	0.7 ± 0.6 (0–2)	1.6 ± 1.5 (0–4)	0.057
Left lower lobe	1.3 ± 1.1 (0–4)	1.1 ± 0.8 (0–3)	1.8 ± 1.4 (0–4)	0.123
**The total CT severity scores**	5.6 ± 4.4 (0–20)	4.1 ± 2.3 (0–11)	8.7 ± 5.8 (1–20)	0.008^**^

Note: Quantitative data were presented as mean ± standard deviation (minimum-maximum), while the counting data were presented as count (percentage of the total). ^*^p < 0.05; ^**^p < 0.01

**Table 3:** The difference of CT severity scores and CT follow-up time between two groups

	Moderate type (number of patients)	Severe and critical type (number of patients)	*P*-value by Mann-Whitney U test
Initial CT	4.1 ± 2.3 (0–11) (*n* = 34)	8.7 ± 5.8 (1–20) (*n* = 16)	0.008^**^
First follow-up CT	4.8 ± 2.4 (1–10) (*n* = 34)	11.3 ± 5.5 (5–19) (*n* = 16)	< 0.001^***^
Second follow-up CT	5.0 ± 2.7 (1–10) (*n* = 28)	12.1 ± 5.5 (7–20) (*n* = 16)	< 0.001^***^
Third follow-up CT	4.9 ± 2.5 (0–9) (*n* = 15)	11.7 ± 5.4 (6–20) (*n* = 15)	0.001^**^
Fourth follow-up CT	4.0 ± 1.6 (0–8) (*n* = 8)	10.6 ± 5.4 (4–20) (*n* = 14)	0.002^**^
Fifth follow-up CT	NA	8.7 ± 5.5 (4–20) (*n* = 8)	NA
Sixth follow-up CT	NA	4.7 ± 0.6 (4–5) (*n* = 4)	NA
Seventh follow-up CT	NA	4.0 ± 0.0 (3–4) (*n* = 2)	NA
Interval-1 (d)	4 ± 2 (2–10)	4 ± 2 (1–8)	0.518
Interval-2 (d)	9 ± 3 (5–13)	8 ± 3 (4–13)	0.102
Interval-3 (d)	14 ± 4 (9–23)	12 ± 3 (8–18)	0.251
Interval-4 (d)	17 ± 2 (14–19)	17 ± 4 (12–26)	0.743
Interval-5 (d)	NA	21 ± 3 (19–26)	NA
Interval-6 (d)	NA	25 ± 7 (18–36)	NA
Interval-7 (d)	NA	29 ± 4 (24–32)	NA

Note: Quantitative data were presented as mean ± standard deviation (minimum-maximum), Interval-1, the interval between initial CT and first follow-up CT; Interval-2, the interval between initial CT and second follow-up CT, and so on. NA, not applicable; d, day

^**^p < 0.01; ^***^p < 0.001

**Fig. 1:**
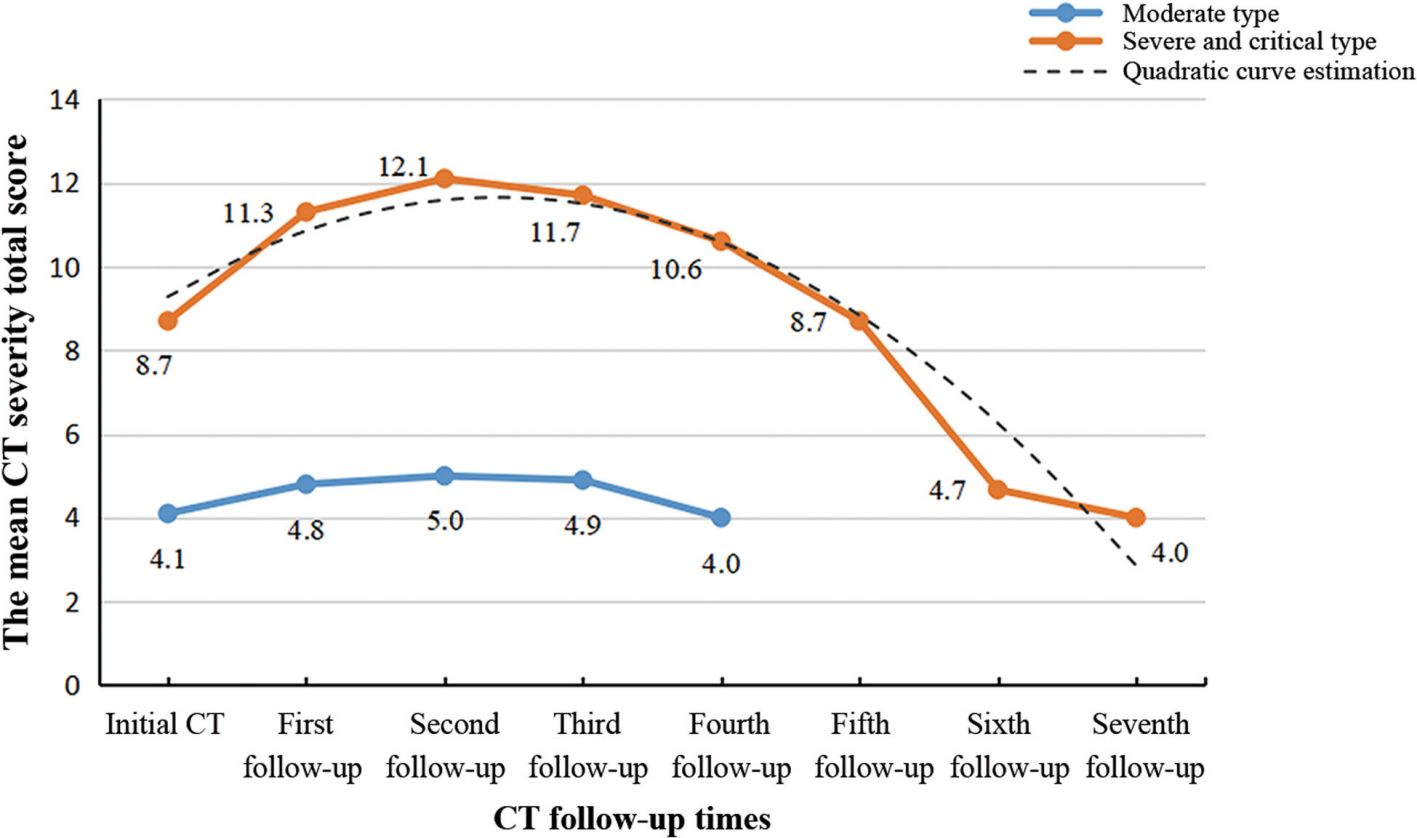
The dynamic changes of total CT severity score with CT follow-up times. In the severe and critical type, peak total CT severity scores occurred at the second follow-up CT (curve fitting equation: y = −0.477*x^2^ + 3.167*x + 6.887, in which x = number of CT examination, y = total CT severity scores; r^2^ = 0.137, *p* = 0.008). In the moderate type, there was no correlation between the total CT severity score and CT follow-up times by the SPSS curve estimation module (*p* = 0.426)

There was a quadratic curve between the total CT severity score and CT follow-up times in the severe and critical group by the SPSS curve estimation module (r^2^ = 0.137, *p* = 0.008, Fig. 1). The total CT severity score in the severe and critical group peaked at the second follow-up CT (12.1 ± 5.5, range 7 to 20) with a mean interval of 8 ± 3 days after the initial CT scan, and then gradually decreased. In the moderate group, there was no correlation between the total CT severity score and CT follow-up times by the SPSS curve estimation module (*p* > 0.05, Fig. 1).

### The findings of CT characteristics in the two groups

Among 50 patients, the occurrence rate of CT characteristics were GGO (88%), pleural thickening or adhesion (52%), crazy-paving pattern (50%), consolidation (48%), fibrosis (32%), discrete nodules (8%), bronchiectasis (6%), thoracic lymphadenopathy (4%), pleural effusion (4%), and cavitation (2%) (Table 2). There was no significant difference in the occurrence rate of all ten CT characteristics in the initial CT between the two groups (all *p* > 0.05). However, there were differences in the occurrence rate of ground-glass opacity (50% vs. 82%, *p* = 0.040) and crazy-paving pattern (75% vs. 39%, *p* = 0.031) in the second follow-up CT, and pleural thickening or adhesion (100% vs. 65%, *p* = 0.019) in the third follow-up CT between the severe and critical group and the moderate group (Table 4). There were no differences in the occurrence rate of consolidation and fibrosis in every follow-up CT between the two groups (all *p* > 0.05, Table 4).

**Table 4:** The findings of top five CT characteristics in the CT follow-up between two groups

CT follow-up	ground glass opacity	consolidation	crazy-paving pattern	pleural thickening or adhesion	fibrosis
**First follow-up CT**					
Moderate type (*n* = 34)	31 (91%)	20 (59%)	15 (44%)	22 (65%)	17 (50%)
Severe and critical type (*n* = 16)	13 (81%)	11 (69%)	11 (69%)	12 (75%)	7 (44%)
*p* value	0.370	0.549	0.135	0.533	0.767
**Second follow-up CT**					
Moderate type (*n* = 28)	23 (82%)	22 (79%)	11 (39%)	22 (79%)	15 (54%)
Severe and critical type (*n* = 16)	8 (50%)	13 (81%)	12 (75%)	16 (100%)	8 (50%)
*p* value	0.040^*^	1.000	0.031^*^	0.072	1.000
**Third follow-up CT**					
Moderate type (*n* = 17)	12 (71%)	8 (47%)	6 (35%)	11 (65%)	9 (53%)
Severe and critical type (*n* = 15)	10 (67%)	11 (73%)	10 (67%)	15 (100%)	10 (67%)
*p* value	1.000	0.166	0.156	0.019^*^	0.491
**Fourth follow-up CT**					
Moderate type (*n* = 8)	7 (88%)	1 (13%)	2 (25%)	6 (75%)	4 (50%)
Severe and critical type (*n* = 15)	10 (67%)	7 (47%)	7 (47%)	14 (93%)	11 (73%)
*p* value	0.369	0.176	0.400	0.269	0.371
**Fifth follow-up CT**					
Severe and critical type (*n* = 8)	3 (38%)	4 (50%)	4 (50%)	7 (88%)	5 (63%)
**Sixth follow-up CT**					
Severe and critical type (*n* = 5)	5 (100%)	0 (0%)	0 (0%)	3 (60%)	3 (60%)
**Seventh follow-up CT**					
Severe and critical type (*n* = 3)	3 (100%)	0 (0%)	0 (0%)	3 (100%)	1 (33%)

^*^p < 0.05

**Fig. 2:**
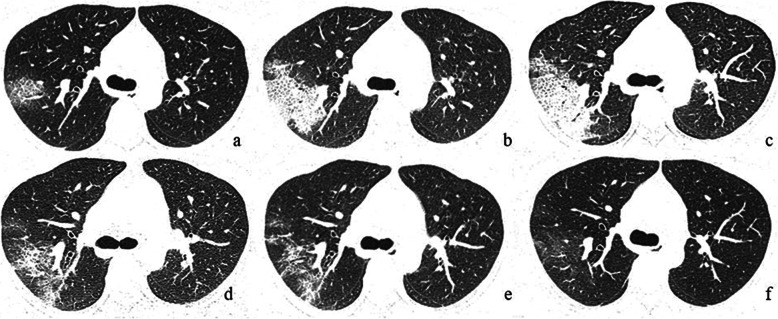
The CT changes of a patient with fever (38.5 °C) for one-day (severe type). **a** At initial CT, a small region of subpleural ground-glass opacity (GGO) was demonstrated in the right upper lobe; **b** the first follow-up (day 4), there was an enlarged region of GGO with superimposed inter- and intralobular septal thickening (crazy-paving pattern) with partial consolidation; **c** the second follow-up (day 8), crazy-paving pattern extended to more regions with a new area of subpleural consolidation; **d** the third follow-up (day 12), partial resolution of the crazy-paving pattern and consolidation; **e** the fourth follow-up (day 17), continued absorption of residual crazy-paving pattern with the presence of pleural adhesion and fibrosis; **f** the fifth follow-up (day 23), minimal residual GGO were observed. All images have the same window level of −700 and window width of 1000

In the severe and critical group, the GGO, fibrosis, and pleural thickening or adhesion could be found in every follow-up CT and were the main signs in the last two CT follow-up (Fig. 2). The proportions of patients with the crazy-paving pattern (75%) and consolidation (81%) reached a peak with the decrease in the proportion of GGO (50%) at the second follow-up CT. The occurrence rate of pleural thickening or adhesion (100 and 100%) peaked at the second and the third follow-up CT.

In the moderate group, the proportions of patients with GGO and the crazy-paving pattern gradually decreased from the initial CT. The proportions of patients with the consolidation (79%) and pleural thickening or adhesion (79%) reached the peak with the decrease in the proportion of crazy-paving pattern (39%) at the second follow-up CT. Both in the third and fourth follow-up CT, the GGO and pleural thickening or adhesion the main demonstration with the obvious absorption of consolidation and crazy-paving pattern, and fibrosis was still present.

## Discussion

In our study, we analyzed the different characteristics and involvement severity of CT in the two clinical groups at initial diagnosis and follow-ups. Our study found that the lesion’s involvement of the severe and critical group in the area of the right lung and whole lung was more extensive than those of the moderate group. The dynamic changes of severity as CT follow-up time were different between the two groups. There were no differences in the occurrence rate of CT characteristics in the initial CT between the two groups, which indicates that the initial CT characteristics had no certain specificity and regularity in distinguishing the severity of COVID-19 pneumonia. However, the extent of pneumonia involvement by CT findings can help clinicians to assess the severity of COVID-19 in the early stage, so as to achieve the purpose of early and accurate treatment.

The study found that the severe and critical group showed more extensive and severer involvement than those in the moderate group, mainly involving the right lung in the initial CT and the whole lung in the initial and follow-up CT. This finding is similar to previous studies [20, 23], which also reported the initial CT score of the whole lung in the severe and critical patients was higher than that in the moderate patients. Furthermore, we found that the patients with severe and critical type progressed rapidly with the greatest severity at the second follow-up CT, and then gradually recovered. The moderate type is relatively stable, though the total CT severity scores also peaked at the second follow-up CT. These findings indicated that the extent of pneumonia involvement by CT could help in the evaluation of the severity and extent of COVID-19 pneumonia. It is also helpful for clinicians to identify and prevent the condition of severe and critical patients from getting worse in the early stage.

In our study, the most common initial CT characteristics of COVID-19 pneumonia of the two groups are ground-glass opacity, crazy-paving pattern, consolidation, pleural thickening or adhesion, and mainly distributed in a subpleural area. These findings were consistent with previous studies [13, 14, 24–26]. Besides, we did not find differences in the incidence of above CT characteristics between the two groups in the initial CT, which was consistent with the previous study [27]. However, the occurrence rate of the crazy-paving pattern in the severe and critical group was higher than that in the moderate group in the second follow-up CT with a mean interval of 8 ± 3 days after the initial CT scan, while the occurrence rate of ground-glass opacity was the opposite. These findings indicated that the progression of the crazy-paving pattern might represent further infiltration of the lung parenchyma and lung interstitium [24, 28]. The previous studies [26, 29, 30] have reported that in the progression or peak period of pneumonia (1–3 weeks), the progression of crazy-paving pattern, septal thickening and consolidation can be observed. However, COVID-19 pneumonia has different CT manifestations at different stages, which are mainly related to pathogenesis. Therefore, the dynamic changes of different clinical types can be demonstrated by CT characteristics, but it is still difficult to distinguish the clinical types by initial CT characteristics.

This study has several limitations. Firstly, the sample size was relatively small, especially in the severe and critical group. Further studies with more patients are warranted to obtain a definitive answer. Secondly, the number and interval of CT follow-up per patient were different, though the interval time of each follow-up chest CT was of no difference between the two groups. These factors need to be considered when interpreting our findings. Finally, the quantitative and semi-quantitative measurements of the pulmonary lesions may have certain subjectivity, and can only partly reflect the severity. Accurate quantitative analyses should be made to identify the changes in clinical and imaging characteristics in future studies.

## Conclusions

In conclusion, the results of this study confirmed the great significance of chest CT for the differentiation of moderate type and severe or critical type, and the dynamic changes of chest follow-up CT in different severity of COVID-19 pneumonia. COVID-19 pneumonia with severe and critical type progressed rapidly with the greatest severity at the second follow-up CT, and the moderate type is stable. There were no differences in the incidence of ten CT characteristics between the two groups in the initial CT. Therefore, the CT changes of COVID-19 pneumonia with different severity were different, and the extent of pneumonia involvement by CT finding can help to evaluate the severity of COVID-19 pneumonia rather than the initial CT characteristics.

## Data Availability

The datasets used and analyzed during the current study are available from the corresponding author on reasonable request.

## Abbreviations

COVID-19: Coronavirus Disease 2019
WHO: World Health Organization
RT-PCR: Reverse transcription-polymerase chain reaction
SpO2: Oxygen saturation
PaO2: Partial arterial oxygen pressure
FiO2: Fraction of inspired oxygen
ICU: Intensive care unit
GGO: Ground glass opacity
